# Metal Sulfide Semiconductor Nanomaterials and Polymer Microgels for Biomedical Applications

**DOI:** 10.3390/ijms222212294

**Published:** 2021-11-14

**Authors:** Athandwe M. Paca, Peter A. Ajibade

**Affiliations:** School of Chemistry and Physics, University of KwaZulu-Natal, Private Bag X01, Scottsville, Pietermaritzburg 3209, South Africa; 217076639@stu.ukzn.ac.za

**Keywords:** semiconductor nanomaterials, metal sulfide, therapeutic agents, drug delivery, polymer microgels

## Abstract

The development of nanomaterials with therapeutic and/or diagnostic properties has been an active area of research in biomedical sciences over the past decade. Nanomaterials have been identified as significant medical tools with potential therapeutic and diagnostic capabilities that are practically impossible to accomplish using larger molecules or bulk materials. Fabrication of nanomaterials is the most effective platform to engineer therapeutic agents and delivery systems for the treatment of cancer. This is mostly due to the high selectivity of nanomaterials for cancerous cells, which is attributable to the porous morphology of tumour cells which allows nanomaterials to accumulate more in tumour cells more than in normal cells. Nanomaterials can be used as potential drug delivery systems since they exist in similar scale as proteins. The unique properties of nanomaterials have drawn a lot of interest from researchers in search of new chemotherapeutic treatment for cancer. Metal sulfide nanomaterials have emerged as the most used frameworks in the past decade, but they tend to aggregate because of their high surface energy which triggers the thermodynamically favoured interaction. Stabilizing agents such as polymer and microgels have been utilized to inhibit the particles from any aggregations. In this review, we explore the development of metal sulfide polymer/microgel nanocomposites as therapeutic agents against cancerous cells.

## 1. Introduction

The search for new therapeutic agents to treat cancer is an active research area because cancer remained one of the public health problems in the world. In the search for novel therapeutic agents, development of nanomaterials has become one of the most effective platforms to fabricate novel therapeutic agents and delivery systems to treat cancer [1]. This is mostly due to the high selectivity of nanomaterials for cancer cells that is associated with the porous nature of tumour cells which allows nanomaterials to accumulate more in tumour cells than in normal cells. Nanomaterials are low dimensional structures with at least one dimension less than 100 nm which confers on these material’s unique properties. These materials could be classified as one dimensional, two dimensional or three dimensional, based on their size and the number of dimensions that are quantum confined [2]. Nanomaterials result from nanotechnology which is a combination of technology and science that functions at nanoscale level to provide novel properties as well as novel applications. The principle of nanotechnology is the capability to design and engineer materials and frameworks in the nanometer range with novel properties for unique applications.

As the particle size gets smaller, the surface-to-volume ratio gets larger and leads to an increase in the rate of any reactions which take place on the surface of the materials as more surface atoms are subject to reaction. Moreover, as particles become smaller, the extent of particles on the surface increases similar to those inside, which results in extraordinary properties for the as-prepared materials [3,4,5]. In addition, as the size of the nanomaterial decreases, quantum effects start to regulate the behaviour and inherent properties of the materials. This is a result of limited electron motion as electron become spatially restrained and give rise to electronic and thermodynamic properties that diverge from those of the mass structure [4]. The physical and chemical attributes (such as chemical reactivity, absorption, emission, and biological mobility) are transformed when the material dimensions move toward the de Broglie wavelength. However, as the size of particles become smaller, the resulting nanomaterials possess striking optical and electronic properties which lead to innovative applications in a wide spectrum of fields.

Morphological properties like size and shape play a huge role in the properties of nanomaterials. The size and particle dispersal are of paramount importance in the electronic, thermodynamic, and chemical properties of the material. The particle size of material influences the structure, lattice parameters, and crystalline nature of any nanomaterial. Nanomaterials with smaller particle size have larger surface area [6]. In the course of synthesis of nanomaterials, the synthetic approach used dictates the shapes (spheres, cubic, tubes, rods, etc.) of the resulting materials [7]. Shapes of nanomaterials are significant in biomedical application such as chemotherapy [8]. Properties such as colour and transparency are a consequence of the optical properties. These properties normally change at a nano-level since nanomaterials are so small that the movement if the electrons in them is restricted, which results in nanomaterials reacting differently with light as compared to bulk materials. Metal sulfide nanomaterials have been investigated owing to their different crystalline structures [9]. Metal sulfide nanomaterials involve sulfur atoms chemically bonded to metal ions [10]. They represent a major group of minerals with various structural phases with the general formula M_x_S_y_ [11,12]. Metal sulfides are naturally versatile compounds compared to metal oxides due to the smaller electronegativity of sulfur compared to oxygen [13]. Thus, it is necessary to review the impact of polymer-based microgel semiconductor metal sulfide nanomaterials as diagnostic tools and therapeutic agents which may offer new opportunities for application in cancer therapy.

## 2. Synthesis of Nanomaterials

The structural attributes of nanomaterials are the most important properties that influences the functional properties and applications of the materials. In order to get the distinctive properties such as the different sizes, shapes, and compositions of nanomaterials, one need to use good synthetic approaches that will give the as-prepared nanomaterials unique properties. Various procedures have been used to prepare nanomaterials and some of them are described below:

### 2.1. The Sol-Gel Method

Small molecules are combined to produce solid nanomaterial with varying shapes. This involves two different phases, namely solution and gelatin (solid) which can be easily separated [14]. This method permits the formation of solid materials and can be utilized to fabricate nanomaterials in different morphologies for various applications [14]. This approach can yield nanostructured materials with high purity and uniform size distributions at low temperatures [15]. This method is mostly used to prepare metal oxide nanomaterials [16,17,18,19]

### 2.2. Hydrothermal/Solvothermal Method

In this method, the synthesis of the nanomaterials is performed in sealed vessel in which the temperature of the solvents can be brought to around their boiling points through heating simultaneously under adequate pressures. This process is called hydrothermal when water is used and solvothermal when organic solvents are used in the synthesis [20].

### 2.3. Vacuum Deposition Method

This method requires an inert gas in vacuum chamber for the preparation of the nanomaterials. During preparation, the temperature of the substrate gets lowered down to −196 ° (77.15 K). Through thermal evaporation, the momentum of the vaporized metallic atoms can be reduced by gas to prevent their agglomeration on the substrate. The evaporated metal atoms get deposited on the layers of the evaporation chamber and the particle sizes can be effectively regulated between 3∼100 nm depending on the reaction conditions such that the temperature of the substrate, evaporation speed, gas pressure and the nature of the gas utilized [15].

### 2.4. Ball Milling Method

This method requires no solvent, it employs simple grinding of the powders together till the required nano-sized alloy is obtained [21]. Hard and fragile ceramic structures can be processed into nanomaterials to give nanocrystals and pseudo-crystals. An addition of approximately 2% of an alcohol can inhibit the dispersion and solid reaction of the nanoparticles [15].

### 2.5. Thermolysis

**Scheme 1:**
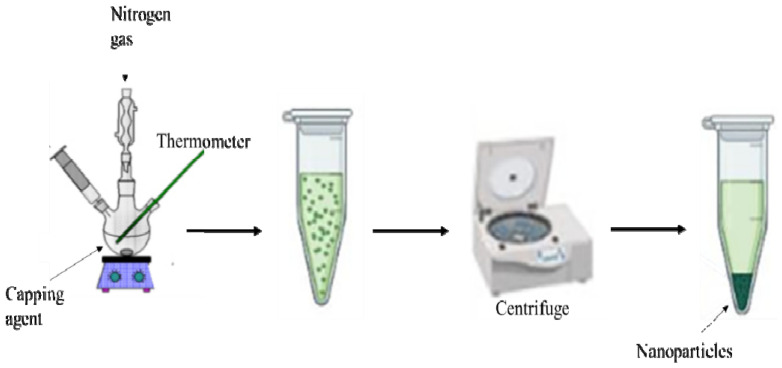
Schematic diagram of the thermolysis process.

This method is acknowledged for the preparation of metal sulfide nanoparticles. The morphology and purity of the nanoparticles can be regulated by varying the reaction conditions such as the nature of the single-precursor molecule [22,23,24], decomposition temperature [25,26], duration of the thermolysis and capping agents [27,28]. Briefly, during this process (Scheme 1), a single-source precursor molecule dispersed in a particular solvent gets injected into a high boiling point coordinating solvents (capping agents) in an inert environment [29]. This method is quite useful as it permits simple control of the relative intensities of the excitonic shortwave against the surface state longwave emission [30]. Khan et al. [31] synthesized spherically-shaped HgS nanoparticles by the thermal decomposition of Hg(II) dithiocarbamate complexes. Arandhara and colleagues [32] thermolyzed ZnS nanoparticles and varied the thermolysis temperature to investigate the generated changes in the growth process. They discovered that at temperatures above 200 °C, thin films with high crystallinity were produced. Ain et al. [33] prepared highly crystalline octylamine- capped CuS nanoplates by thermolysis of Cu(II) dithiocarbamate. The authors noted that these nanoplates have good catalytic efficiency and photocatalytic performance.

## 3. Nanomaterials in Biomedicine

**Figure 1:**
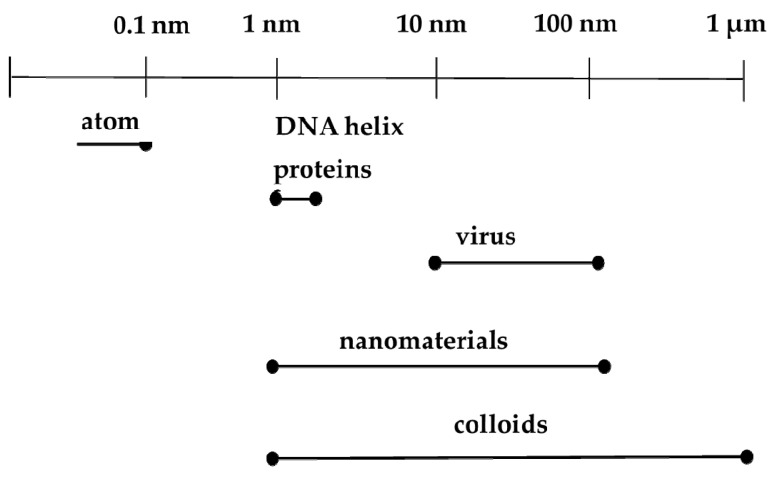
Comparison of nanoparticles and biological units.

**Figure 2:**
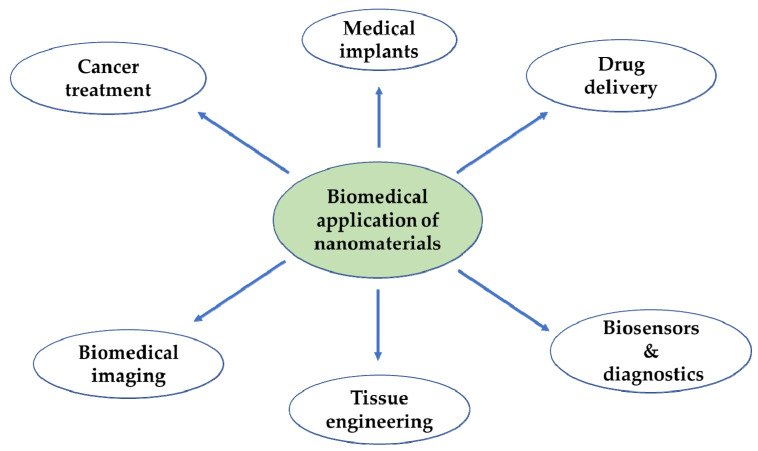
Application of the nanomaterials in the biomedical field.

In the context of biomedicine, nanoparticles could improve the intracellular concentration of drugs in tumorous cells without any effect on normal cells. They normally do this by bypassing the P-glycoprotein, the main drug resistance mediator [34]. Another advantage is that vital building blocks of life such as DNA and proteins fall within the nanometer range (Figure 1). For instance, double stranded DNA has intra-strand with spacing of approximately 2 nm while cell membranes have a thickness of about 10 nm. Haemoglobin has a diameter around 5.5 nm and insulin is about 3 nm in diameter [35]. New therapeutic and diagnostic nanomaterials fabricated with at least one dimension in the nanometer range exhibit physicochemical properties that induce host reactions at a biomolecular level that are different from those of their bulk forms. They provide notable advantages over conventional systems such as improved and regulated delivery, prolonged drug bioactivity, enhanced circulation times and greater dissolution rates [36]. These nanomaterials are at present being used for different biomedical applications as shown in Figure 2.

### Metal Sulfide Nanomaterials in Cancer Therapy

These are semiconductor materials with various applications in biomedicine that are attributable to their distinctive properties at the nanometer scale, such as their absorption in the infrared region which result in a narrow optical band gap, high fluorescence, structural and thermal steadiness [37,38], water-dispersibility, non-toxicity, exceptional catalytic and photocatalytic activity and electric conductivity [39,40]. To date, these semiconductor nanomaterials are being developed for therapeutic applications due to their peculiar physical and chemical attributes [41]. Typically, metal sulfide semiconductor materials are perfect species for the development of chemotherapeutic agents owing to their robust near-infrared (NIR) absorbance, minimal toxicity and good photochemical degradation [42]. Metal sulfide nanomaterials have been fabricated for the therapeutic and diagnosis of cancer. They have been used as diagnostic tools in photoacoustic [43,44] and multimodal imaging [45,46]. Photothermal therapy [47], photodynamic therapy [48], radiotherapy [49] and chemotherapy [50] for cancer treatment. Metal sulfide nanomaterials are promising frameworks in cancer photothermal therapy because of their elevated absorbance in the NIR region.

**Figure 3:**
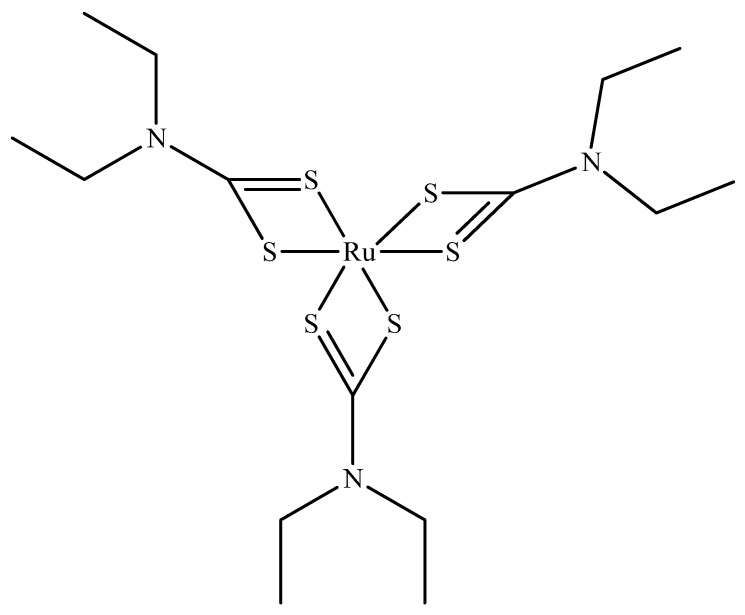
Tris(diethyldithiocarbamato)ruthenium(III) ([Ru(DDTC)_3_]).

**Figure 4:**
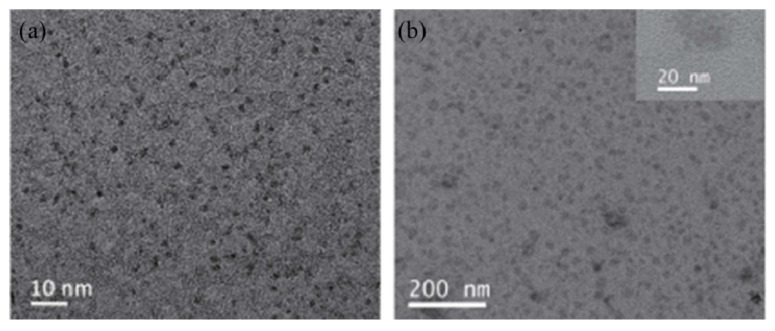
TEM images of RuS_1.7_ nanodots (**a**) and PEG-dBSA-RuS_1.7_ nanoclusters (**b**) [53].

Ruthenium sulfide is among the group of transition metal chalcogenides that are being explore as potential therapeutic agents [51,52]. Lu et al. [53] prepared novel photothermal agents, ruthenium sulfide-based (PEG-dBSA-RuS_1.7_) nanoclusters. Firstly, RuS_1.7_ nanodots were prepared by the decomposition of tris(diethyldithiocarbamato)ruthenium(III) ([Ru(DDTC)3]) (Figure 3) in oleic acid and ethyl alcohol. The resulting as-prepared nanorods showed a uniform particle size with an average particle size of 1.5 nm (Figure 4a). These nanodots were then coated with denatured bovine serum albmin (dBSA) and poly(ethyleneglycol) (PEG) to form water soluble nanoclusters with an average size of 23 nm as illustrated in Figure 4b, a suitable size for photothermal application. These photothermal agents exhibited great physiological stabilities, notable photothermal properties on NIR laser irradiation, outstanding biocompatibility, and very low toxicity. Noteworthy, the in vivo chemotherapeutic studies showed that these nanomaterials have a prolonged blood circulation and tumour accumulation in comparison to other photothermal agents.

Xie and colleagues [54] designed dual-function tin sulfide (SnS) nanosheets and applied them in cancer theranostics. They synthesized the nanosheets from bulk SnS and functionalized them with targeting and imaging agents. The SnS nanosheets exhibited good biocompatibility and effective photothermal activity. Moreover, steady photothermal performance over brief times of laser irradiation and photodegradability over extended exposure was observed. As a result of their morphology, they showed high loading of small molecules that confirm their capability as drug carrier. The in vitro and in vivo experiments showed improved anticancer activity with no noticeable side effects and toxicity to healthy cells.

Lu et al. [55] fabricated a stimuli-responsive nanocomposites made up of copper sulfide (CuS) nanoparticles and graphene oxide (GO) nanosheets to act as carrier for the distribution of doxorubicin (DOX) for thermal chemotherapy. The nanoparticles acted as delivery system for DOX molecules and GO nanosheets prevented the leakage of drug and improved photothermal energy conversion efficacy. The CuS-DOX/GO nanocomposites were fabricated in such a way that they discharge the DOX in response to pH and near-infrared light stimuli, making the therapeutic effect to be well-regulated which led to high specificity. The in vitro cytotoxicity against HeLa cells proved that the CuS-DOX/GO nanocomposites are potential therapeutic and delivery systems. Hou and associates [56] devised a dual-function tumour targeted CuS nanoparticles which could serve as drug carrier and photoacoustic imaging agent to direct photo-chemotherapy. Cytotoxic evaluation showed that the system sustained a high intracellular drug concentration within tumour cells, resulting in improved therapeutic effect.

## 4. Drug Delivery

Drug delivery refers to the administration of pharmacological drugs into the body to accomplish its anticipated therapeutic effect. Drug delivery has been broadly studied. It involves a wide spectrum of techniques used to administer therapeutics in the body [57]. The most important trait of any drug delivery material is its aptitude to control the release, minimalize the side effects and expand the therapeutic efficiency of drugs [58]. Kheirandish and colleagues [59] prepared ruthenium sulfide nanoparticles encapsulated in chitosan (RuS_2_-NPs-CS) nanocomposites as an effective in vitro delivery system for curcumin and antibacterial agents. The results revealed that incorporation of RuS_2_ nanoparticles into chitosan provided a potential drug carrier with high drug loading and release efficiency. These nanocomposites also showed antibacterial activity against *Pseudomonas aeruginosa* (PAO-)1 bacterial.

The use of polymers as drug delivery system can curb many of the drawbacks posed by free therapeutic agents, such as poor stability, solubility, biodistribution and pharmacokinetics, rapid degradation and non-specific toxicity [60,61]. Drug delivery systems can be constructed to afford regulated drug release with minimum unfavourable side effects [62,63]. Polymers play a vital role in our everyday lives. A polymer is a molecule consisting of several repetitive subunits. Our bodies are made up of biomolecules such as proteins and nucleic acid which are in fact polymeric. All biological processes are regulated by many factors because of changes in stimuli. Synthetic polymers that are stimuli responsive find application in biomedicine due to their capability to mimic biomolecules. They are used as therapeutic agents, biosensors, and drug delivery systems especially for cancer. Synthetic polymers show better pharmacokinetics such as longer circulation time and reduced toxicity [64]. Polymers are also better drug delivery systems because of their tunable properties which allow regulated release of encapsulated drug within the polymer.

Drug delivery to patients is best physiologically suitable way that has constantly been a major challenge for a very long time [65]. Better consideration of changes in the environment of pathological tissues, has enabled for the development of new materials capable of reacting particularly to specific “peculiar” conditions of affected tissues [66]. Some polymers can undergo particular changes in their physiochemical properties as a reaction to changes in their surroundings, that can be manipulated to design stimuli-responsive delivery systems [66]. Thermo-responsive polymeric systems have been used to design temperature responsive drug delivery systems which have critical transition temperature [67]. Since, tumour cells are normally distinguished by higher temperature than the normal cells, the temperature difference may possibly help to formulate thermos-responsive drug delivery systems [68].

### 4.1. Polymer Microgels as Drug Delivery Systems

Among synthetic polymers, microgels has emerge as the most interesting group of polymers. Microgels are units of gel with a diameter of about 50 nm to 10 µm. They are cross-linked macromolecules with high molecular weight and possess a globular shape. Microgels exhibit various significant properties such as tunable structure, porous nature and modifiable dimensions [69]. They have an ability to undergo rapid volume phase transitions. In other words, they can swell and de-swell through different environmental inducement such as change in temperature, ionic strength, and pH. They can be easily synthesized and the synthetic technique used influences essential properties such as the size and functionality, which are significant, specifically for regulating drug loading and release dynamics, durability, biodistribution and biocompatibility, degradation and bioaccumulation, and functionality in the perspective of a drug delivery purposes [70]. Microgels are currently being investigated and find applications in cosmetics, surface coatings, catalysis, and drug delivery. Polymer microgels have emerged as good delivery vehicles for metal nanoparticles due to their distinct properties that are suitable for the conditions of drug vehicles used via systematic routes of administration [71]. Microgels have high water content and desirable mechanical properties. The size of microgel is tunable from nanometers to micrometers hence they are significant tools for polymer-based delivery frameworks [72].

Microgels have received tremendous attention for the development of drug delivery system. This is because of their capability to deliver therapeutic agents particularly to tumorous tissues and consequently reduce side effects that result from unintended delivery to healthy tissues [65]. Also, aqueous microgel are highly hydrophilic with low interfacial energy in a biological medium which result in and lowered nonspecific interactions with proteins and accumulative bioavailability and biocompatibility. The core microgels permits the potential integration of fairly big quantities of drugs [65].

Microgels offer a convenient to control the surface morphologies which could be used to maneuver the structures at a nanometer to micrometer scale [73]. This enables the incorporation of other novel materials with specific functional groups to control their dispersity within the microgel [73,74]. Microgels offer various advantages such as easy preparation, tunable degree of cross-linking, colloidal stability, and stimulus response. These properties allow incorporation of nanoparticles within the microgel. Incorporation of nanoparticle can be achieved by three methods [75]. The first method uses the microgel as a template for in situ preparation of inorganic nanoparticles [76]. The second method involves diffusion of the as-prepared nanoparticles into the microgel [77]. The third method involves the fusion of the as-prepared nanoparticles with reactive surfaces in microgels straight for the duration of the synthesis. This route result in effective incorporation and rigid bonding of the nanoparticles by covalent bonds in microgel system to inhibit nanoparticle outflow [78].

**Figure 5:**
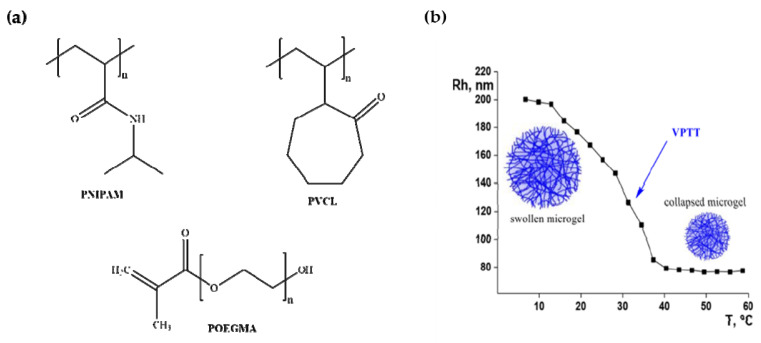
(**a**) Structures of thermos-responsive polymers. (**b**) Illustration of volume phase transition of PNINAM.

Microgels made from poly(*N*-isopropylacrylamide) (PNIPAM) [79], poly(vinylcaprolactam) (PVCL) [80] and poly(oligo(ethyleneglycol)methacrylate) (POEGMA) [81] shown in Figure 5a, have lower critical solution temperature (LCST) in aqueous medium and precipitate from solution when heated to a temperature beyond their LCST. PNIPAM was first synthesized in the 1950s and is the most common thermos-responsive polymer used in drug delivery systems [82]. PNINAM exhibits reversible phase transition upon heating and becomes poorly soluble in water as the temperature of the solution rises. It has a distinct structure and its thermal response is close to that of the human body [83,84]. It has LCST of 32 °C, and at any temperature lower than this, it becomes hydrophilic and water soluble due to the hydrogen-bonding interactions which results in a coil-like conformation. Above the LCST, it becomes very hydrophobic and forms a viscous gel which stick to tissues. PNIPAM has been used to prepare microgel for therapeutic targets such as in drug targeting for tumour, in drug delivery and in tissue engineering [82,85]. PNIPAM microgels are acknowledged drug delivery systems as they tend to shrink above their volume-phase transition temperature (VPTT) and release any molecules present within the microgel (Figure 5b) [86].

Core-shell microgels consist of a hydrophobic core and a hydrophilic shell. The characteristics of the core and the shell may be combined to produce decent drug delivery systems. Metal-containing microgels are being developed as therapeutic and diagnostic tools. They have been employed as the main templates for the preparation of metal nanomaterials incorporated into polymer matrix [71]. Wu et al. [58] prepared core-shell hybrid nanogels consisting of Ag nanoparticles as core and poly(*N*-isopropylacrylamide-*co*-acrylic acid) (PNIPAM-*co*-AA) gel as shell and investigated the cell imaging and pH-controlled drug delivery with optimum drug stacking capability. PNIPAM-*co*-AA gel shell can expand and shrink because of pH adjustment. This manipulates the properties of the incorporated nanoparticles and modifies the size of the gel shell to control the way in which the drug is being discharged. pH-responsive microgels possess properties such adjustable size, large surface area for conjugation of biomolecules, internal network arrangement for integration of drugs, reduced undesired impacts of drugs and regulated drug release at a precise pH surrounding [72]. Several pH-responsive polymer microgels have been developed and incorporated with bioactive molecules such as drugs for regulated release [87,88,89]. The pH-responsive hybrid microgels are tunable delivery systems for specific purposes when they go through local stimuli in pursuit of the affected area such as tumour cells that are characterized by alterations in acid or base equilibrium [90,91,92].

Muratalin and colleagues prepared functionalized PNIPAM microgels using the emulsion polymerization synthetic route. The microgels showed stimuli response to changes such as change in temperature, pH and introduction of copper(II) ions and glucose. The stimuli response of the microgels and VPTT which is similar to that of the human body made these microgels suitable drug delivery platforms that can be controlled by external stimuli [93]. Recently, Khan and co-workers reported a multi-responsive poly(*N*-isopropylacrylamide-allyl acetic acid) (PNIPAM-AAA) copolymer microgel loaded with CuO nanoparticles. The results demonstrated that the hybrid microgels filled with nanoparticles possess notable antibacterial activity [94]. Zhang et al. [76] synthesized nanoparticles using microgels as a template and investigated their magnetic properties. The results showed that the properties of nanoparticles depend on the framework and structural hierarchy of microgels, and conditions used for the preparation. These hybrid microgels have promising applications in catalysis, medicine, and chemical separation technologies.

### 4.2. Synthesis of Microgels

The synthesis of microgels involves regulating some important characteristics properties for the microgel to be able to perform significant functions. It is important to regulate the size and particle size dispersity of microgel particles throughout the preparation process. Thermo-responsive microgels are mostly made by precipitation and inverse emulsion polymerization [95].

#### Precipitation Polymerization Method

This method is the generally preferred synthetic route for the preparation of aqueous microgels. It is mostly used for the preparation of thermo-sensitive microgels [96]. It regulates the extent of cross-linking, functionality, and the microgel size and size distributions. Complex compounds such as core-shell microgels, can be formulated by using metallic nanoparticles or microgels as nucleation points equivalent to the parent particles [70]. During precipitation polymerization, an aqueous solution of the monomer, cross-linker and surfactant gets heated beyond the LCST of the polymer, and then a free radical initiator is added [70]. The resultant polymer chain collapses since the reaction temperature is much beyond the VPTT and precipitates from solution to form a parent molecule which grows via aggregation with other phase-separated polymer chains to form microgels [97]. When polymerization is completed, the mixture is left to reach room temperature, the microgel system swells and attain a “furry” morphology. At temperature under the VPTT, microgels are sterically stabilized owing to the presence of hydrogen bonds between the polymer fragments and water molecules [86,96]. This method has been extensively employed for the preparation of thermosensitive PNIPAM [97,98,99,100,101,102,103,104,105,106,107,108,109,110,111] and poly(N-vinylcaprolactam) (PVCL) [102].

Karg et al. [104] employed the precipitation polymerization method to prepare gold nanorod-coated PNIPAM microgels. They studied the thermal behaviour of the microgels using an ultraviolet-visible spectrometer. The spectra were measured at 15–50 °C, where the microgels were fully swollen at 15 °C and totally deswollen at 50 °C which suggested the presence of two distinct nanorod loading systems, with low and high surface coverage. From the spectra, they observed a rise in absorbance at low wavelength up to 650 nm, and a red-shift and enhanced longitudinal plasmon band. These effects were correlated with the improved refractive index of the gel during compression of the microgel, which increased the Rayleigh scattering radiation [104,105] and also increased the local refractive index adjacent the Au nanorods [106,107]. In conclusion, their results also confirmed that the VPT of pure PNIPAM microgel is completely reversible.

Das and colleagues [106] also used the precipitation polymerization method to prepare a group of thermo-responsive microgels loaded with gold nanorods and they investigated their thermal-induced volume transitions to establish a framework with an intense transition in the physiologically appropriate range between 38–41 °C in saline buffer solution with a pH of 7.4. They picked the two most optimistic microgels, specifically poly(NIPAm-MA) microgels (series M2) and poly(NIPAm-NIPMAm)/PAA IPN microgels (series IPN4), to investigate the integration of the nanorods in the microgel core. TEM images of the two systems revealed that the nanorods were successfully incorporated and evenly dispersed within the microgels. Secondly, they studied thermal-induced transitions in the size of hybrid microgels and discovered that, at room temperature, series M2 had a smaller diameter (by about 7%), than the pure microgels, which was ascribed to the higher ionic strength of the hybrid material and cross-linking of the microgel moiety with the nanorods. When the temperature was raised, the hybrid microgel collapsed to smaller size. The VPTT took place in the speculated range of 38–41 °C and the VPTT of the hybrid system and the pure microgel was very similar. A similar trend was observed for hybrid poly(NIPAm-NIPAm)/PAA microgels but the degree of contraction of the hybrid microgel was considerably less than that of the pure microgel. They also investigated the photothermally induced VPT of the hybrid systems. The microgels were expose to radiation at λ = 809 nm and experienced great reversible, photothermally induced volume transition. These two microgels are potential candidates for photothermally regulated drug release.

### 4.3. Hybrid Metal Sulfide-Microgels

Extensive research has been conducted in the design of hybrid metal sulfide microgels. The combination of the microgel and metal sulfide materials may offer novel compounds with a vast array of advantages [73]. The use of naturally occurring and biocompatible polymers as templates for preparation of metal sulfide quantum dots (QDs) presents significant advantages compared to other techniques. Farooq et al. [109] used a gum Arabic (GA) microgel as template for the preparation of metal sulfide quantum dots (QDs). Metal ions were loaded into GA microgels and treated with aqueous sodium sulfide solution. Transmission electron microscopy (TEM) image revealed the metal sulfide QDs were successfully incorporated and evenly distributed within the microgel. Growth of properly shaped QDs crystals with the microgel matrix was observed. Pich et al. reported the preparation of hybrid microgels loaded with ZnS nanomaterials. The prepared hybrid microgels possessed both thermosensitive properties of the polymer (poly(N-vinylcaprolactam-co-acetoacetoxyethylmethacrylate) microgel and physical and chemical attributes of the ZnS material. Powder XRD results revealed low intense peaks for the hybrids which was correlated with the partial crystalline nature and low concentration of the ZnS nanomaterials within the microgel matrix.

### 4.4. Polymer Nanofibers

Nanofibers are fibers with a diameter of less than 100 nm. Nanofibers possess desirable properties that are convenient for biomedical, and biotechnology uses such as tissue engineering frameworks, drug delivery systems, biosensors, medical implants, etc. These properties include their large surface-to-volume ratio, tiny pore size, distinguished porosity, and unique physicochemical and biological properties [109,110]. In addition, nanofibers have large surface than the normal fibers since they have a one-dimensional (1D) structure [111].

Various natural biomaterials such as keratin, collagen, polysaccharide cellulose and chitin, are well ordered fibrous structures that exist at nanometer scale. This makes polymer nanofibers appropriate to provide a suitable method to mimic biosystems. Since cells live in a micro- or nano-featured environment, polymer nanofibers have an ability to regulate cellular actions with regards to cell adhesion, stimulation, proliferation, arrangement and orientation [110]. Polymer nanofibers can be used as drug delivery platforms since they have ability to allow controlled drug release. The dissolution rate of a drug and the surface area of the encapsulated drug have a direct relation [112].

Yang et al. [113] developed doxorubicin-encapsulated active-targeting micelles in poly(vinyl)alcohol/gelatin core-shell nanofibers for drug delivery. These electrospun nanofibers afforded a prominent therapeutic efficiency by means of minimum drug dosage, yet the systematic drug subjection to normal tissues was effectively diminished. Shao et al. [114] fabricated poly(ɛ-caprolactone)/multi-walled carbon nanotubes composite nanofibers loaded with green tea polyphenols (GTP). The nanofibers showed reduced cytotoxicity for healthy cells, however, inhibition effect to tumour cells was high which suggested that this material might be a good candidate for cancer treatment.

#### 4.4.1. Fabrication of Nanofibers

Several fabrication methods such as electrospinning, template synthesis, drawing, phase separation and self-assembly have been employed to make appropriate polymer nanofibers for different purposes [109,115]. Although there are several routes used to prepare nanofibers, electrospinning remains the most popular technique for nanofiber preparation [110]. Electrospinning is a renowned procedure used to produce continuous, one-dimensional (1D) fibers with diameters varying between nanometers (nm) to micrometers (μm) [116,117]. During the process, a polymer solution gets spun at high voltage through electrostatic charging [117,118,119]. It is very cost effective, has high production rates and is straightforward. This technique offers homogenous and lengthy fibers with various morphologies such as a hollow, rigid and core-shell chemical frameworks [120]. These fibers exhibit incredibly high surface-to-volume ratios which makes them perfect carriers for the controlled delivery of pharmaceutical agents such as anticancer drugs, antibiotics, some vitamins and growth factors [110].

#### 4.4.2. Formation of Nanofiber by Electrospinning

In the general process, a polymer solution (contained in a syringe pump) pushed through a syringe needle and a grounded target is subjected to an electric field. A spurt gets discharged from the surface of the charged polymer solution when the applied voltage and electrostatic repulsion counteracts the surface tension which results in the construction of a so-called Taylor cone [121]. When the voltage surpasses a certain limit, the electric force overpowers the surface tension of the droplet and then the charged spurts of the solution are expelled from the tip of the droplet depending on the intensity of the electric field. As the jet travels in the direction of a collecting counter electrode, the solvent evaporates and results in the formation of a fabric-like mat [122,123].

The morphology and diameter of electrospun nanofibers depend on several electrospinning parameters. (1) Solution conditions such as viscosity, conductivity surface tension and elasticity [124]. It was established that solution viscosity influences the diameter of the fiber [125], morphology [126] and the jet path [127]. Also, polymer solution with low viscosity result in electrospraying, meaning instead of getting fibers, small particles are produced [128]. Beachley et al. [129] discovered that the length, diameter and uniformity of polycaprolactone (PCL) nanofibers increases with increasing polymer viscosity. (2) Processing conditions such as applied voltage, needle diameter, tip-target length and feeding rate of the polymer solution [122,130,131]. (3) The environmental variables such as the temperature, humidity and static electricity. The humidity and temperature of the surrounds may influence the morphology and diameter of the fibers [132]. Casper et al. [133] investigated the impact of humidity and solution viscosity on the surface of electrospun polystyrene fibers. When the fibers were electrospun in an environment with a humidity below 25%, uniform fibers with no pores on the surface were obtained, but with a humidity greater than 30%, pores started forming on the surface of the fibers. Therefore, increasing humidity resulted in occurrence of thick and scattered pores on the fibers. In addtion, an increase in the molecular weight of the polystyrene yielded thicker and irregular shaped fibers with larger pores.

Kehren et al. [111] prepared homogenous poly(vinyl alcohol) (PVA) and poly(*N*-vinylcaprolactam) (PVCL) microgel microfibers using the electrospinning technique. They investigated the solution parameters to decide the best conditions to achieve fibers free of defects and defined morphology. Different PVA/microgel solutions were electrospun and discovered which shows that at lower PVA concentrations, no defined fibers were formed. Whereas with concentrated polymer solution, defined morphologies were obtained. They discovered that the polymer solution viscosity and flow rate play a vital role in the production of the fibers. The properties of the microgels were investigated by differential scanning calorimetry (DSC) and thermos-gravimetric analysis (TGA). DSC was used to evaluate the volume phase transition of the microgel-based fibers which proved that the microgel particles undergo reversible VPT. The swelling properties of the microgels and fibers were determined by TGA. The results revealed that the thermos-response of the microgels is sustained within the fibers. Toxicity evaluations revealed that the microgels and the fibers are non-toxic, which implied that they can be used for biomedical applications.

## 5. Conclusions

Semiconductor metal sulfide nanomaterials have been synthesized, characterized, and applied in various fields; however, very little information is available with regards to their medical applications. The approaches used for the preparation of metal sulfide nanomaterials as well as their use as drug delivery agents, diagnostic tools and probably therapeutic agents are of great interest. The main shortcoming of these materials lies in their poor water solubility; however, they possess some other fascinating properties such as size-dependency, more binding sites and photothermal properties which could enhance their application in the biomedical field. Stimuli-responsive microgels are promising drug delivery platforms. Their high-water solubility and biocompatibility make them good templates for the design of hybrid microgels which can be loaded with metal sulfide nanomaterials for drug delivery purposes. Since metal sulfide nanomaterials have poor solubility, encapsulating them within a microgel matrix might be a solution. This could lead to the development of potent therapeutic agents in the future.
